# Diagnostic performance of a novel loop-mediated isothermal amplification (LAMP) assay targeting the apicoplast genome for malaria diagnosis in a field setting in sub-Saharan Africa

**DOI:** 10.1186/s12936-015-0926-6

**Published:** 2015-10-09

**Authors:** Eniyou C. Oriero, Joseph Okebe, Jan Jacobs, Jean-Pierre Van geertruyden, Davis Nwakanma, Umberto D’Alessandro

**Affiliations:** Medical Research Council Unit, PO Box 273, Banjul, The Gambia; Institute of Tropical Medicine, Antwerp, Belgium; International Health Unit, University of Antwerp, Antwerp, Belgium; Department of Microbiology and Immunology, KU Leuven, Leuven, Belgium; London School of Hygiene and Tropical Medicine, London, UK

**Keywords:** Malaria diagnosis, LAMP, RDT, Microscopy, PCR, Field-based, Resource-limited settings

## Abstract

**Background:**

New diagnostic tools to detect reliably and rapidly asymptomatic and low-density malaria infections are needed as their treatment could interrupt transmission. Isothermal amplification techniques are being explored for field diagnosis of malaria. In this study, a novel molecular tool (loop-mediated isothermal amplification—LAMP) targeting the apicoplast genome of *Plasmodium falciparum* was evaluated for the detection of asymptomatic malaria-infected individuals in a rural setting in The Gambia.

**Methods:**

A blood was collected from 341 subjects (median age 9 years, range 1–68 years) screened for malaria. On site, a rapid diagnostic test (RDT, SD Bioline Malaria Antigen P.f) was performed, thick blood films (TBF) slides for microscopy were prepared and dry blood spots (DBS) were collected on Whatman^®^ 903 Specimen collection paper. The TBF and DBS were transported to the field laboratory where microscopy and LAMP testing were performed. The latter was done on DNA extracted from the DBS using a crude (methanol/heating) extraction method. A laboratory-based PCR amplification was done on all the samples using DNA extracted with the Qiagen kit and its results were taken as reference for all the other tests.

**Results:**

*Plasmodium falciparum* malaria prevalence was 37 % (127/341) as detected by LAMP, 30 % (104/341) by microscopy and 37 % (126/341) by RDT. Compared to the reference PCR method, sensitivity was 92 % for LAMP, 78 % for microscopy, and 76 % for RDT; specificity was 97 % for LAMP, 99 % for microscopy, and 88 % for RDT. Area under the receiver operating characteristic (ROC) curve in comparison with the reference standard was 0.94 for LAMP, 0.88 for microscopy and 0.81 for RDT. Turn-around time for the entire LAMP assay was approximately 3 h and 30 min for an average of 27 ± 9.5 samples collected per day, compared to a minimum of 10 samples an hour per operator by RDT and over 8 h by microscopy.

**Conclusion:**

The LAMP assay could produce reliable results the same day of the screening. It could detect a higher proportion of low density malaria infections than the other methods tested and may be used for large campaigns of systematic screening and treatment.

## Background

Malaria control programmes aiming at decreasing local transmission and achieving pre-elimination status require proper detection of *Plasmodium* parasites in the peripheral blood, both in febrile patients and asymptomatic carriers [1]. Asymptomatic carriers, representing the human reservoir contributing to the transmission of the parasites, generally have much lower parasite densities compared to symptomatic individuals, at detection thresholds (<200 parasites/μL) below which the standard diagnostic tools such as microscopy and rapid diagnostic tests (RDTs) become less reliable [2, 3]. For malaria elimination efforts to have a better chance of success, it is crucial to identify as many malaria-infected carriers as possible in order to treat them and possibly interrupt transmission. Therefore, the ideal diagnostic tool to support malaria elimination efforts should have high sensitivity to detect most if not all infected individuals [1].

Molecular methods, such as polymerase chain reaction (PCR), reliably detect low-grade and asymptomatic infections from different sample types, including dried blood spots (DBS) [4, 5]. However, it is difficult to use PCR in field settings because of the equipment and infrastructure required [6]. New molecular diagnostic tools such as loop-mediated isothermal amplification (LAMP) have been developed to facilitate rapid target amplification through single-temperature incubation, thus reducing the facility and equipment requirement compared to PCR-based methods [7]. LAMP has a potential for use in point-of-care (POC) diagnosis of malaria and is already being tested in clinical and field settings [8–13].

The development and validation of a novel LAMP assay targeting the apicoplast genome of *Plasmodium falciparum* has previously been reported and it showed comparable sensitivity and specificity, when tested with archived DNA samples, as compared to standard PCR method targeting the 18srRNA locus [14]. The objective of this study was to evaluate the diagnostic performance (sensitivity, specificity and predictive values) and operational characteristics (turn-around time and ease of use) of this novel LAMP assay in a field setting. Results of the LAMP assay were compared with those obtained using a standard laboratory-based PCR assay.

## Methods

### Study area and participants

This study was carried out as part of the screening stage of an ongoing trial in the eastern part of The Gambia, as published elsewhere [15]. Briefly, individuals from the study villages around the Basse field station of the Medical Research Council (MRC) Unit in The Gambia were screened between October and December 2014. Verbal consent was obtained prior to screening, after a careful explanation of the information sheet in the local language. All individuals with a fever (body temperature ≥37.5 °C) were excluded. Ethical clearance was obtained from the Gambian Government/MRC Joint Ethics Committee in The Gambia (approval number: SCC1321 and L2014.55).

### Sample collection and processing

From a single finger prick, blood samples were collected for RDT (SD Bioline Malaria Antigen P.f, HRP 2, Ref: 05FK50), thick blood film and dried blood spots—DBS (Whatman^®^ 903 Specimen collection paper LOT 6833909/82). The RDTs were read immediately in the field while the DBS and microscopy slides were taken to the field station at the end of each day for further processing. Slides from RDT positive individuals were stained and read immediately upon arrival in the laboratory at the field station, according to the study protocols of the main trial; those from RDT negative individuals were stained the same day but read later for this study. Thick blood films were stained with 10 % Giemsa for 10 min and examined under 1000-fold magnification by trained microscopists. Asexual and sexual parasite density was determined by counting the number of parasites per 500 white blood cell (WBC) and parasite density was estimated by assuming a mean WBC count of 8000/μL. All slides were double read and a third microscopist resolved all discrepancies.

About a quarter of the DBS samples were processed in the field station for LAMP, to determine the turn-around time, while the rest were analyzed later with the same methods. DNA was extracted from the DBS using a crude methanol and heating extraction method, immediately followed by the LAMP assay. In addition, DNA was also extracted from the same DBS samples using a QIAamp DNA mini kit (Qiagen GmbH, Hilden, Germany) and used in a laboratory-based PCR assay that was taken as the reference method.

### DNA extraction for the LAMP assay

Two 3-mm discs of the DBS were punched for each sample into a 96-well Round-Well Block (Qiagen) using a 96-well plate plan having the corresponding sample screening and identification numbers. A hundred and fifty µl of methanol were added to each well and incubated at room temperature for 10 min. The methanol was removed and the punched discs dried at room temperature for approximately 45 min before adding 75 µL of distilled water. The punched discs were mashed using a new pipette tip for each well and the Round-Well Block was heated in a water bath at 95 °C for 10 min. The supernatant was then transferred to a fresh 96-well plate and 3 µL of this from each sample was used for the LAMP assay.

### LAMP assay

**Fig. 1:**
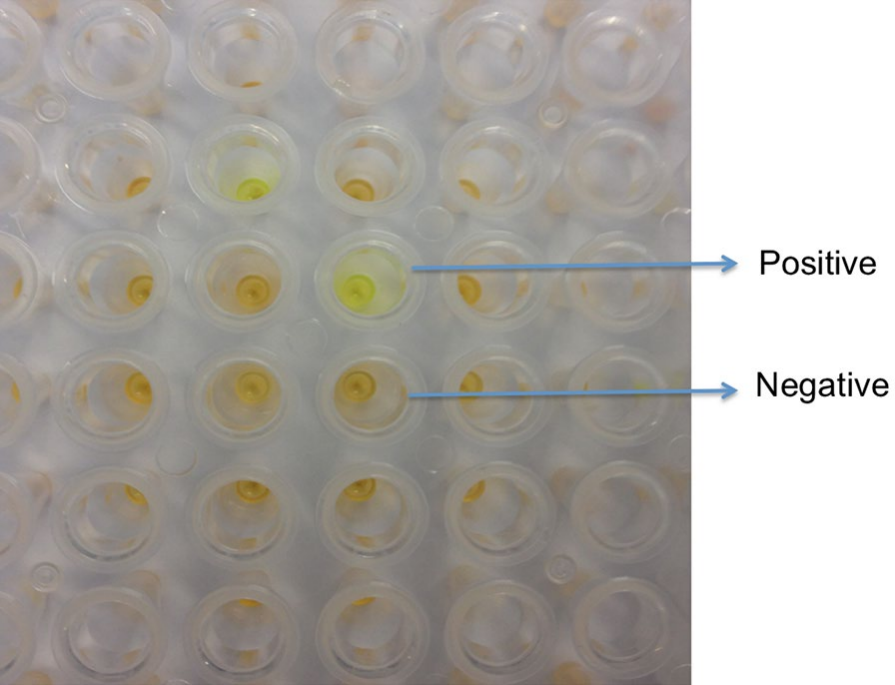
Visualization of LAMP reaction in a 96-well plate format showing colour differences between positive and negative samples

The final amplification reaction mixture (25 µL) was carried out as previously described [14] and contained 1.6 µM each of the inner primers FIP and BIP, 0.2 µM each of the outer primers F3 and B3c, 0.8 µM each of the loop primers LPF and LPB, 1× Isothermal Amplification Buffer, 1 Unit of Bst 2.0 WarmStart™ DNA polymerase (New England Biolabs), 1.0 mM of each dNTPs, 1.0 mM MgCl_2_, and 3 µL of DNA sample. Known positive and negative controls were included in each run. The reaction was performed at 65 °C for 60 min in a water bath and the end product was determined and verified visually, after addition of SYBR Green I, (naked eye) for color change by two blinded operators (Fig. 1).

### Reference PCR assay

A laboratory-based PCR amplification was done for all the samples using DNA extracted with the Qiagen kit, according to protocols published by Snounou et al. for both pan genus and species-specific amplification [16]. End product of the PCR amplification was visualized and double-scored with ethidium bromide-stained agarose gel electrophoresis.

### Sample size calculation and data analysis

Sample size was calculated using G*Power version 3.1 [17]. A total number of 317 samples were required at 95 % power, 5 % precision, an effect size of 0.25, and 5 degree of freedom (df). The performance characteristics of the LAMP assay as well as that of microscopy and RDT were calculated using the standard PCR assay as the reference method. Cohen’s kappa (κ coefficient) was also calculated to assess the degree of agreement between the results from the different tests. Comparison of the area under the receiver operating characteristic (ROC) curves in a paired design was used to assess the relative accuracy of each of the tests. Precision of the estimates was determined by calculating 95 % confidence interval (CI) (rounded up to the nearest whole number) for each test statistic. Statistical analysis was performed with Stata 13 (StataCorp, College Station, TX, USA).

## Results

During the study period, 528 samples were collected, with a mean of 27 ± 9.5 individuals screened daily. This analysis includes 341 samples for which the results for all diagnostic tests performed are available. The median age of the individuals screened was 9 years, ranging from 1 to 68 years; there were more females (200/341; 59 %) than males (141/341; 41 %), and most study subjects (78 %) were less than 15 years old (Table 1). Malaria prevalence by microscopy was 30 % (104/341), and more than half (59 %) had parasite densities ≤200 parasites/μL. Prevalence as determined by RDT (126/341; 37 %) and LAMP (127/341; 37 %) did not differ. There were more discrepancies between tests at parasite densities <200 parasites/μL.

**Table 1:** Age groups, gender (%) and parasite density of individuals screened

Age group (years)	Male	Female	Total	Median parasite density (range)
<5	38 (11 %)	54 (16 %)	92 (27 %)	192 (16–9120)
5–14	81 (24 %)	94 (28 %)	175 (52 %)	64 (16–41,280)
≥15	22 (6 %)	52 (15 %)	74 (21 %)	64 (16–65,600)
Total	141 (41 %)	200 (59 %)	341	

### Diagnostic performance characteristics

**Fig. 2:**
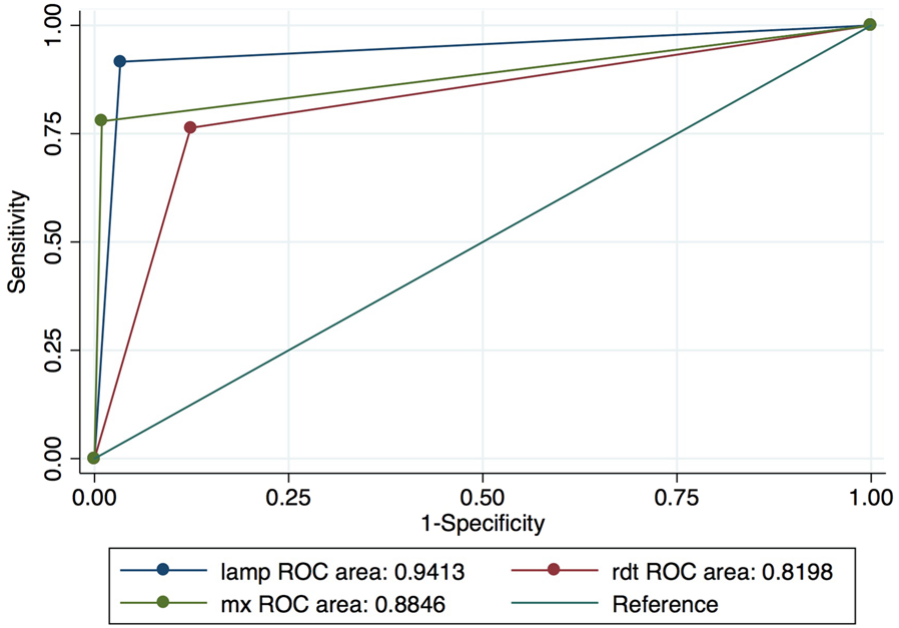
AUC ROC curve comparison against the reference PCR method. Analysis was done using STATA 13 (StataCorp, College Station, TX, USA). *mx* microscopy, *RDT* rapid diagnostic test, *lamp* loop-mediated isothermal amplification

LAMP had a sensitivity of 92 %, a specificity of 97 %, and positive predictive value (PPV) and negative predictive value (NPV) of 95 %. Microscopy had a sensitivity of 78 %, a specificity of 99 %, a PPV of 98 % and NPV of 88 %. Sensitivity, specificity, PPV and NPV for RDT were 76, 88, 79 and 86 %, respectively. The sensitivity of LAMP was significantly higher than those observed by either microscopy or RDT (exact McNemar significance probability <0.0001); κ coefficient was 0.9 for LAMP, 0.8 for microscopy and 0.6 for RDT (Table 2). Area under the ROC curve for the LAMP assay was 0.94 while that of microscopy and RDT were 0.88 and 0.82, respectively (Fig. 2).

**Table 2:** Comparison of all the tests against the reference PCR method

	PCR +ve	PCR −ve	Total	
LAMP +ve	120	7	127	Sn = 91.6 % (95 % CI 85.5–95.7); Sp = 96.7 % (95 % CI 93.3–98.7)
LAMP −ve	11	203	214	PPV = 94.5 % (95 % CI 89.1–97.8); NPV = 94.9 % (95 % CI 91.1–97.4)
				κ = 0.91 (95 % CI 0.84-0.94)
Mx +ve	102	2	104	Sn = 77.9 % (95 % CI 70.1–84.7); Sp = 99.1 % (95 % CI 96.6–99.9)
Mx −ve	29	208	237	PPV = 98.1 % (95 % CI 93.2–99.8); NPV = 87.8 % (95 % CI 82.9–91.7)
				κ = 0.80 (95 % CI 0.73-0.87)
RDT +ve	100	26	126	Sn = 76.3 % (95 % CI 68.1–83.3); Sp = 87.6 % (95 % CI 82.4–91.8)
RDT −ve	31	184	215	PPV = 79.4 % (95 % CI 71.3–86.1); NPV = 85.6 % (95 % CI 80.2–90.1)
				κ = 0.64 (95 % CI 0.56-0.73)
Total	131	210	341	

*Sn* sensitivity, *Sp* specificity, *κ* Kappa coefficient, *PPV* positive predictive value, *NPV* negative predictive value

### Operational characteristics

It took approximately three and half hours each day to process samples for the LAMP assay, which is similar to a minimum of 10 samples an hour, per operator by RDT in comparison with over 8 h in total for the same number of samples by microscopy. The time required for the drying and incubation steps of the crude DNA extraction method, together with the total run-time of the LAMP assay represented the largest amount of time used in the LAMP test, from the time the samples arrived at the facility to the results (Table 3). All the incubation steps were done either at room temperature or in a water bath so no extra equipment was needed. The LAMP assay was high throughput and there was perfect agreement of visual scoring of the results by naked eye between the two independent readers.

**Table 3:** Processing and turn-around-time of the LAMP assay for the number of samples collected daily (mean of 27 ± 9.5)

Process	Time used (h)
Punching of DBS samples including decontamination after each sample	1
DNA extraction	1.25
LAMP assay and end point determination	1.25
Turn-around-time	3.5

## Discussion

This study shows the feasibility of deploying LAMP testing in a field setting using a novel assay developed in-house, with diagnostic performance characteristics >90 % when compared with a laboratory-based reference PCR method. Previous work comparing the LAMP assay with a nested PCR assay using archived DNA samples also showed similar results [14]. The diagnostic performance of the LAMP assay was also similar to that of a commercial LAMP testing kit used for detection of asymptomatic malaria parasite carriers in Zanzibar, in comparison with RDT and real-time PCR [13]. Although a comprehensive cost analysis was not done in this study, it is expected that in-house developed assays would be cheaper than commercial kits [18].

The relatively high malaria prevalence is not surprising as the sample collection was carried out during the peak of the malaria season, in the eastern part of The Gambia, where transmission is still relatively high. Previous studies carried out in the same region have reported malaria prevalence determined by molecular methods ranging from 14 to 26 %, while this was much lower by microscopy [19, 20].

Although all individuals with fever were excluded during the screening stage, information on recent history of fever or malaria infection was not obtained and this may explain the higher prevalence and low agreement observed with RDT, which detects circulating histidine-rich protein II (HRP 2). Malaria indicator surveys (MISs) carried out in four countries (Kenya, Senegal, Zambia, and Mozambique), reported that prevalence determined by RDT was higher than by microscopy. Possibly, recently treated infections and persistent circulating antigen (up to 6 weeks after infection clearance, in the absence of an ongoing infection) [21, 22] may be responsible for the high rate of false positives observed with the HRP-2 based RDT. Though the prevalence determined by RDT was similar to that by LAMP, the former had a higher number of false positives, suggesting that carrying out systematic screening with RDT followed by treatment of those tested positives would unnecessarily treat an important proportion of individuals while others with asymptomatic infections would go undetected.

Processing the samples in a 96-well plate format with the LAMP assay was easy to perform and would reduce both processing and handling time considerably, compared to processing smaller batches in individual tubes. When comparing this with other formats for end point determination of the amplified products, such as agarose gel electrophoresis, visualization of color change by naked eye was an easier and faster method, reducing post amplification manipulations. Overall, it would be much easier to train staff to use and interpret the results with the LAMP assay than with standard PCR.

## Conclusion

With a shorter turn-around-time and high throughput processing, LAMP results would be available much faster than by microscopy and more reliably than with RDT, supporting the use of this diagnostic test for mass screening and treatment (MSAT) or focused screening and treatment (FSAT) campaigns. The apicoplast genome that this novel LAMP assays targets is unique to the parasite, thus ensuring high specificity. There have been recent improvements in the use of LAMP for field diagnosis, which includes the use of lyophilized or freeze-dried reagents and quick one-step DNA extraction methods or use of direct blood. Although molecular methods have only recently been recommended for surveillance or parasite detection in low-endemic regions, as the move for global malaria elimination continues, simplified molecular tools such as LAMP will most likely become the favoured option for diagnosis of asymptomatic malaria infections.
